# When prophylaxis turns pathologic: a case of LMWH-induced necrosis with secondary cellulitis

**DOI:** 10.1093/omcr/omaf271

**Published:** 2025-12-26

**Authors:** Mohammed AbuBaha, Bara AbuBaha, Hossam Salameh, Asem Afana, Ibrahim Abseh, Hatem Taha

**Affiliations:** Department of Medicine, An-Najah National University, Rafidia Street, Nablus P400, Palestine; Department of Medicine, An-Najah National University, Rafidia Street, Nablus P400, Palestine; Department of Medicine, An-Najah National University, Rafidia Street, Nablus P400, Palestine; Department of Medicine, An-Najah National University, Rafidia Street, Nablus P400, Palestine; Department of Medicine, An-Najah National University, Rafidia Street, Nablus P400, Palestine; Head, Department of Internal Medicine, Palestine Medical Complex, Al Ma'ahed Street, Ramallah P601, Palestine; Faculty of Medicine and Health Sciences, An-Najah National University, Rafidia Street, Nablus P400, Palestine

**Keywords:** dermatology, allergy, immunology, pharmacology and pharmacy

## Abstract

**Introduction:**

Low-molecular-weight heparins (LMWHs) are widely used during pregnancy and postpartum to prevent thrombosis and are generally safe. Rarely, they can trigger delayed hypersensitivity reactions causing skin necrosis and infection. Recognizing this complication is important for timely care.

**Case presentation:**

A 40-year-old woman developed painful necrotic patches at enoxaparin injection sites on her abdomen and arm one week after cesarean delivery. The lesions were red, tender, and associated with elevated inflammatory markers, while platelet counts were normal, excluding heparin-induced thrombocytopenia. She was diagnosed with LMWH-induced delayed hypersensitivity complicated by cellulitis. Enoxaparin was stopped, and she recovered with apixaban, intravenous antibiotics, wound care, and partial debridement.

**Discussion:**

These reactions usually appear 5–14 days after starting LMWH and can mimic infection or thrombosis. They reflect T-cell–mediated vascular injury and require clinical attention.

**Conclusion:**

LMWH-induced skin necrosis is rare but serious. Early recognition and switching to alternative anticoagulation are essential for favorable outcomes.

## Introduction

Delayed-type hypersensitivity (DTH) is a rare but significant complication of low-molecular-weight heparin (LMWH), characterized by necrotic skin lesions. Immune-mediated reactions usually occur 5–14 days after LMWH initiation and present as painful, erythematous plaques that may progress to necrosis at injection sites [1, 2]. The underlying mechanism involves a type IV hypersensitivity reaction, with T-cell-mediated inflammation causing vascular injury and tissue necrosis [1].

LMWHs are widely used for thromboprophylaxis in medical, surgical, and obstetric settings due to their safety profile [3]. Severe cutaneous reactions are uncommon (<0.1%) but can be underrecognized and present diagnostic challenges. Risk factors may include obesity, female sex, and prolonged heparin exposure, though precise predisposing mechanisms remain unclear [2, 4]. Histopathology typically shows lymphocytic infiltrates, endothelial swelling, and microthrombosis, distinguishing this condition from heparin-induced thrombocytopenia (HIT) [5].

Management involves immediate discontinuation of the causative agent, switching to alternative anticoagulation (fondaparinux or direct oral anticoagulants), and, in selected cases, immunosuppressive therapy [5].

## Case presentation

A 40-year-old woman (G3P1) presented to the emergency department with painful, dark skin lesions that developed over previous enoxaparin injection sites approximately one week after an elective cesarean section at 37 weeks for suspected fetal distress. Her postpartum course had initially been uncomplicated, and she had been discharged on oral antibiotics and ibuprofen. She had received prophylactic enoxaparin (60 mg daily) throughout pregnancy due to advanced maternal age and a history of two early miscarriages. She had no known thrombophilia or autoimmune conditions.

**Figure 1:**
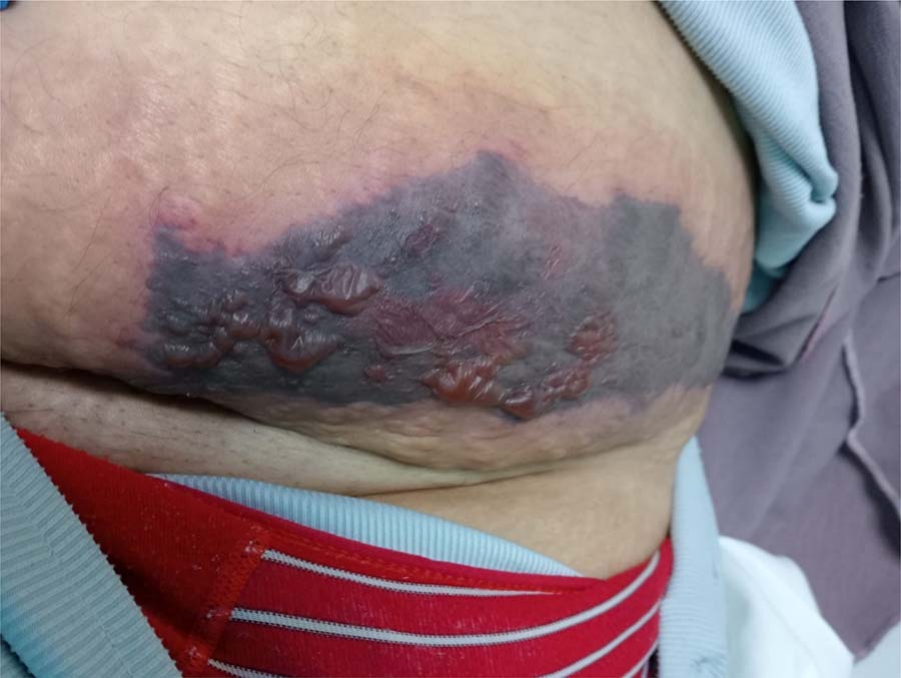
Necrotic plaque with hemorrhagic bullae over the lower abdominal enoxaparin injection site.

**Figure 2:**
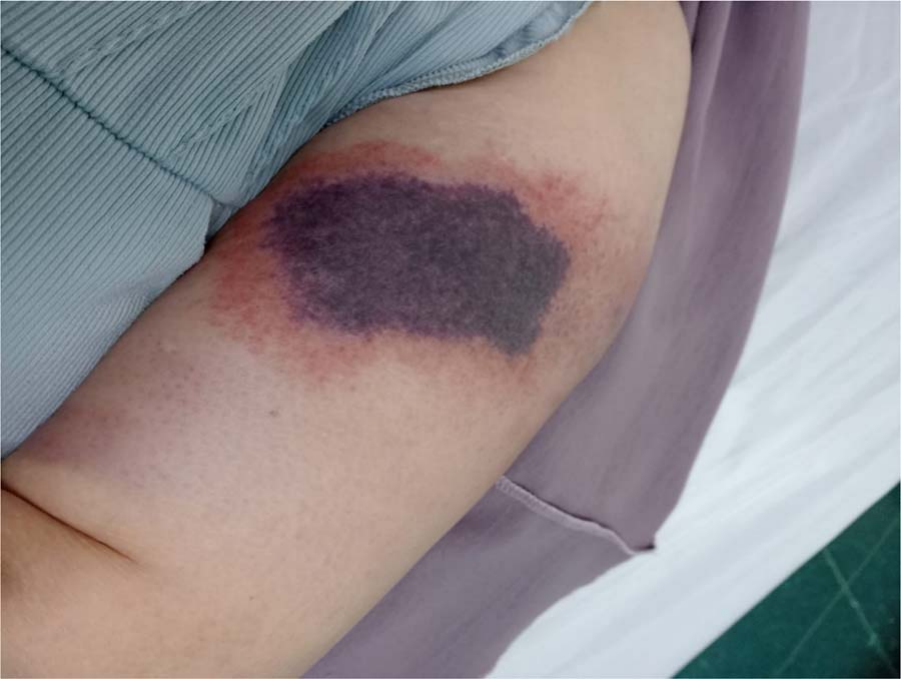
Dark necrotic patch with erythema on the upper left arm at another injection site.

On examination, she was hemodynamically stable and afebrile. Two well-demarcated necrotic plaques were noted: one on the lower abdomen, featuring hemorrhagic bullae, and another on the left arm, presenting as a dark necrotic patch with surrounding erythema and tenderness, as seen in Figs. 1 and 2 below.

Laboratory findings revealed elevated C-reactive protein (CRP) and erythrocyte sedimentation rate (ESR), along with mild leukocytosis. Platelet count and coagulation parameters were normal, excluding HIT. The clinical picture was consistent with LMWH-induced delayed hypersensitivity complicated by localized cellulitis.

Management included immediate discontinuation of enoxaparin, initiation of apixaban, intravenous antibiotics, and daily wound care. Partial surgical debridement was performed after one week. Clinical improvement was noted, and she was discharged on oral antibiotics and apixaban, with instructions to avoid Nonsteroidal Anti-Inflammatory Drugs and scheduled outpatient follow-up.

## Discussion

LMWH, particularly enoxaparin, is considered safe during pregnancy and postpartum for thromboprophylaxis [3, 6]. Rarely, delayed hypersensitivity reactions can occur, mimicking infection or thrombotic events, which complicates diagnosis [1, 2, 7]. Cutaneous reactions have been reported in approximately 7.5% of LMWH users, with necrosis being exceptionally rare [4, 8, 9]. These reactions typically appear 5–14 days after initiation and reflect T-cell–mediated vascular injury [1, 4, 8]. Case reports have described enoxaparin-induced panniculitis and necrosis, highlighting the importance of awareness in obstetric populations [10].

Prompt recognition and cessation of LMWH, switching to a non-heparin anticoagulant, local wound care, and antibiotics when infection is suspected are recommended [5]. Our patient’s clinical course aligned with these recommendations, demonstrating that early detection and appropriate intervention result in favorable outcomes.

## Conclusion

Delayed cell-mediated LMWH-induced skin necrosis with secondary cellulitis is rare but potentially serious. Early recognition, discontinuation of LMWH, and switching to alternative anticoagulation are crucial for recovery, particularly in postpartum patients. Increased awareness supports timely diagnosis and better outcomes in similar cases.

## Statements and declarations

Not Applicable.

## Ethical considerations

All procedures performed in this report involving human participants were in accordance with the ethical standards of the institutional, national research committee, and with the 1964 Helsinki declaration and its later amendments or comparable ethical standards.

According to the institutional regulations, no ethical approval was required to publish any information in this case report.

## Consent to participate

Not applicable.

## Ethics statement and consent to publication

The authors obtained verbal and written informed consent from the patient regarding this case and any accompanying images. A copy of the written consent is available for review by the Editor in Chief of this journal on request.

## Supplementary Material

SCARE_Checklist_omaf271

SCARE_Guideline_Checklist_omaf271

## Data Availability

The data is not available to the public due to privacy concerns.
